# Investigating the potential immunomodulatory effects of commercial oral probiotic supplements on equine gastrointestinal tract barrier function

**DOI:** 10.3389/fimmu.2024.1487664

**Published:** 2025-01-21

**Authors:** Agnieszka Żak-Bochenek, Paulina Żebrowska-Różańska, Joanna Bajzert, Łukasz Łaczmański, Bogumiła Szponar, Natalia Siwińska, Klaudia Gładysz, Katarzyna Sikorska, Anna Chełmońska-Soyta

**Affiliations:** ^1^ Department of Immunology, Pathophysiology and Veterinary Preventive Medicine, Faculty of Veterinary Medicine, Wrocław University of Environmental and Life Sciences, Wrocław, Poland; ^2^ Independent Researcher, Canberra, ACT, Australia; ^3^ Laboratory of Genomics and Bioinformatics, Hirszfeld Institute of Immunology and Experimental Therapy, Polish Academy of Sciences, Wrocław, Poland; ^4^ Laboratory of Medical Microbiology, Hirszfeld Institute of Immunology and Experimental Therapy, Polish Academy of Sciences, Wrocław, Poland; ^5^ Department of Internal Diseases and Clinic of Diseases of Horses, Dogs and Cats, Faculty of Veterinary Medicine, Wrocław University of Environmental and Life Sciences, Wrocław, Poland; ^6^ Student Scientific Association, Hirszfeld Institute of Immunology and Experimental Therapy, Polish Academy of Sciences, Wrocław, Poland; ^7^ Division of Phytopathology and Mycology, Department of Plant Protection, Faculty of Life Sciences and Technology, Wrocław University of Environmental and Life Sciences, Wrocław, Poland

**Keywords:** probiotics, horses, biosurfactants, secretory IgA, microbiome

## Abstract

**Background:**

Oral probiotic dietary supplements are widely used in veterinary medicine, including in horses. It is hypothesized that the presence of probiotic strains can both modulate the intestinal microbiota and affect mucosal immunity parameters. Such a study has not yet been conducted in horses.

**Methods:**

This study involved 12 healthy horses, which were randomly divided into a control group and a group that received a commercial oral probiotic formula containing *Lactobacillus rhamnosus*, *Pedioccus acidilactici* or *Enterococcus faecium* for 84 days. Fecal samples were collected from all horses on day 0 (D0), 28 days after starting the probiotic (D28), 56 days (D56), 84 days (D84) and 28 days after stopping the probiotic (DX) treatment. The samples were subjected to microbiome analysis via next-generation sequencing of hypervariable regions V3-V4 and V7-V9 of the *16S rRNA* gene for analysis of short-chain fatty acids via HPLC analysis and fecal secretory immunoglobulin A (SIgA) quantification via ELISA.

**Results:**

Microbiome analysis revealed no significant differences in either alpha or beta diversity parameters between the groups. No probiotic strains were detected in the samples. Significant changes were detected in three taxa: the family *Bacteroidales* RF16 group, the genus *Erysipelotrichaceae* UCG-004, and the genus *Fibrobacter* during the study in both groups. In all the cases, there was a gradual decrease in relative abundance over time. The concentrations of SCFAs, specifically acetic and propionic acids, significantly increased over time in both groups according to the generalized linear mixed effects (GLME) model. There were no significant differences in fecal SIgA secretion.

**Conclusion:**

The present study revealed no effect of the use of a commercial probiotic dietary supplement on either mucosal immunity or the composition of the intestinal microbiota.

## 1 Introduction

Oral supplements containing probiotics are increasingly popular in veterinary medicine, including in horses. According to the WHO definition, a supplement can be considered a probiotic if it contains live microorganisms that, when summed in adequate amounts, confer a health benefit to the host (1). The most commonly used probiotic species in horses include *Lactobacillus* sp., *Bifidobacterium* sp., and *Enterococcu*s sp., *Saccharomyces* spp., which, according to available knowledge, constitute a small percentage of the composition of the intestinal microbiome; however, their use appears to have positive effects (2–4). Probiotics (live cultures, lyophilized cultures or their fermentation products), prebiotics (substrates that are selectively utilized by host microorganisms conferring a health benefit) or postbiotics (Short Chain Fatty Acids (SCFAs), i.e., butyric acid) are used in hippiatry in dietary supplements for supportive effects in the case of diarrhea, antimicrobial treatment, under stress, or antiparasitic treatment (1, 4).

The effect of probiotics on the gastrointestinal tract is primarily to reduce pathogenic bacteria, which occur through immunomodulatory effects, the production of antimicrobial peptides, competitive exclusion, and inactivation of bacterial toxins (1). When they interact with intestinal epithelial cells, they increase mucus production, providing a mechanical barrier and scaffolding for the “mucosal kill zone.” Probiotic bacteria can also interact with the host immune system by releasing biosurfactants, which are surface active agents that exhibit antimicrobial activity. Most studies have investigated the positive effects of biosurfactants released by lactic acid bacteria (5). Probiotics can affect the composition of the microbiome in horses. A single *in vivo* study revealed a positive effect of probiotic use on the composition of the microbiome in foals (6), and a trend was observed for prebiotic effects on the diversity indices of the gastrointestinal (GI) microbiome in adult stallions (7). *In vitro* studies conducted in cultures of intestinal contents revealed no effect of probiotic application at 24 or 48 hours on alpha or beta diversity indices and noted limited taxonomic differences. However, differences were noted in the production of SCFAs, products of bacterial metabolism (8).

The potential immunomodulatory properties of probiotics are based on strengthening the barrier of nonspecific immunity (i.e., Tight junctions between intestinal epithelial cells) but also interact with T regulatory cells and B cells, stimulating them to differentiate into plasmocytes and produce secretory immunoglobulin A (SIgA) (1). The quantification of fecal SIgA may provide useful information about intestinal permeability and how well the intestinal epithelial barrier performs its immune exclusion and inclusion functions (9, 10). Our own investigations revealed a significant relationship between the level of fecal SIgA and the presence of *Ruminococcus* and *Elusimicrobia*, which belong to the *Firmicutes* phylum and are predominant in horses, as well as the diversity factor of the microbiome (3). In the literature, conclusive data on the effects of probiotic supplements on fecal SIgA secretion in mice and humans are lacking (11–14). This may be due to species differences in the individuals studied, the different compositions and qualities of these supplements, and the timing of administration and frequency of fecal investigation. There is a lack of specific data for horses.

The purpose of this investigation was to determine how long-term use of a commercial probiotic supplement in healthy pastured horses affects mucosal immunity (level of fecal SIgA) and the composition of the intestinal microbiota and its ability to produce SCFAs.

## 2 Materials and methods

### 2.1 Materials

The investigation involved 12 horses of different sexes, breeds and ages kept within a single breeding center. To mitigate the influence of maintenance and nutritional factors on outcomes, all horses were maintained collectively within a uniform pasture setting. Their diet primarily comprised hay derived from polyculture offered ad libitum. On average, one kilogram of hay comprised 14% water, 25% fiber, 2.5% fat, 57 grams of protein, 5 grams of calcium, and 2 grams of phosphorus. The bioloigical composition of the hay in this region of the country (as well as the vegetation available in the pasture) was grasses: *Holcus lanatus L*., *Festuca rubra L*., *Phalaris arudinacea L.*, *Arrhenatherum elatius L*., *Alopecurus pratensis L*.; other plants: a *Lotus uliginosus Schkhur*., *Juncus conglomeratus L.*, *Rumex crispus L.*, *Rumex acetosa L.*, *Vicia cracca L*., and others with a proportion of less than 1%. As a dietary supplement, horses could avail themselves of mineral licks containing 30% sodium, 4.60% calcium, 2.36% magnesium, 1.05% phosphorus, and included sodium chloride, magnesium oxide, calcium carbonate, dicalcium phosphate, and molasses (0.3%). Additives per kilogram included 6,000 mg of zinc (oxide), 1,000 mg of manganese (II) oxide, 700 mg of iodine (coated granulated anhydrous calcium iodate), 280 mg of cobalt (coated granulated cobalt (II) carbonate), and 25 mg of selenium (coated granulated sodium selenite). Access to these licks and water was unrestricted and horses self-regulated their intake. Consequently, it was unnecessary to augment their diet with concentrate feed. Additionally, the horses were not subjected to sport or pleasure training, nor engaged in breeding procedures pertinent to reproduction. To facilitate acclimatization, horses remained at the facility for a minimum duration of three months preceding the initiation of the study. During both the acclimatization and study periods, horses did not receive any form of treatment, including antibiotic or pain and anti-inflammatory therapies. All horsess were dewormed routinely with oral pastes containing ivermectin (18.7 mg/g) and praziquantel (140.3 mg/g) at a dose of 1.07 g paste per 100 kg body weight 3 months prior to sampling.

The investigation material consisted of fecal samples collected at 28-day intervals, according to the study trial described in Section “3.2.1”. Horses were brought to individual boxes for sampling to avoid confusion. Fresh fecal samples were sterilely secured at 1 g each into two Eppendorf-type 2 ml tubes, and 1 g was placed in 5 ml of 70% ethanol in a 15 ml Falcon tube. Immediately after collection, the samples were frozen at -20°C and then transferred to -80°C. Four grams of feces were stored at 4°C for parasitological investigation.

### 2.2 Methods

#### 2.2.1 Study trial

The horses were randomly divided into 2 groups of 6 individuals each. The control group included 4 mares and 2 geldings, with an average age of 15 years and an average weight of 632 kg; the study group included 3 mares and 3 geldings, with an average age of 15 years and an average weight of 589 kg. On D0 of the investigation, each horse underwent a gastrointestinal history and a clinical examination, and fecal samples were collected for preventive testing. The horses were then separated into 2 groups, and in the investigation group, the administration of a probiotic supplement dedicated to horses was started orally at a dose of 5 g/100 kg horse per day, according to the manufacturer’s guidelines, for 84 days. The purpose of the probiotic was to support the gastrointestinal tract during the grazing period, according to the manufacturer’s instructions and veterinary indications. The composition of the preparation was as follows: lyophilized bacteria *Lactobacillus rhamnosus* NCIMB 8010 (5.0 x1012 CFU/g), *Pedioccocus acidilactici* NCIMB 8018 (3.8 x 1010 CFU/g) and *Enterococcus faecium* NCIMB 11181 (4.0 x 1010 CFU/g) and lyophilized bacterial fermentation products (biosurfactants) of *Lactobacillus rhamnosus* NCIMB 8010, *Pedicoccus accidilactici* NCIMB 8018, and *Enterococcus faecium* NCIMB 11181 (obtained from a minimum of 5x 1012 CFU of bacteria). Additionally, the formulation includes a sucrose carrier, calcium carbonate, and bentonite. In the study group, the probiotic was administered every morning by placing it in a piece of carrot. Similarly, horses in the control group received carrots without preparation. The study group received the probiotic for a period of 84 days, according to the manufacturer’s and veterinarian indications, as support during the grazing period. Fecal samples were taken on day zero (D0), 28 days after application (D28), 56 days after application (D56), 84 days after application (D84), and 28 days after discontinuation (DX). The samples were collected between December 25 (D0) and April 16 (DX).

#### 2.2.2 Fecal egg count – modified McMaster method

Fecal egg count analysis was performed to establish the number of small strongyle (*Cyathostomum* spp.) eggs via a modified McMaster method, as previously described (15).

#### 2.2.3 Fecal SIgA level estimation via ELISA

For further SIgA analysis, it was necessary to prepare sample solutions. After thawing at room temperature, the fecal samples were prepared as described in the literature, with modifications (3, 16). From wet fecal samples, 1 g (± 0.05 g) of each sample was suspended in 5 ml of phosphate-buffered saline (PBS) solution (pH ~ 7.4). To thoroughly mix and release the proteins, the samples were subjected to the following procedure: shaking (15 min) and resting (3 min), twice. The samples were then centrifuged for 20 minutes at 1600 × g. The resulting centrifugation supernatant was collected, filtered through a cap filter (40 µm) and mixed with an inhibitor cocktail (10 µl/1000 µl of supernatant; Calbiochem Protease Inhibitor Cocktail Set I 539131). The samples were subsequently centrifuged again for 15 minutes at 3260 × g. A total of 600 µL of the supernatant was withdrawn for the protein content to be evaluated, and the excess protein was frozen at -80°C for further analysis. The concentration of IgA immunoglobulin in the feces was determined via indirect ELISA. The ELISA methodology was modified by adapting the test to fecal samples and validating an in-house ELISA that was compatible with a commercial test (ab190530 horse IgA ELISA Kit). Microplates (Nunc Maxisorp; Thermo Scientific) were coated with polyclonal goat anti-horse IgA H&L antibodies (Abcam, ab112868; 5 µg/mL in 0.05 M carbonate buffer, pH 9.6; 100 µL per well; incubation conditions, 2 hours at room temperature). The plates were subsequently washed five times and blocked with Tris-buffered saline (TBS; 50 mM Tris; 0.14 M NaCl; pH=8.0) containing 0.05% Tween-20 (Sigma−Aldrich; TBST/200 μL per well). The plates were incubated overnight at 4°C. Fecal samples were diluted 1:5 with sample diluent (SD; Abcam, ab190530). A standard IgA horse calibrator (Abcam, ab190530) was used. The final concentrations of the calibrator ranged from 500–7.8 ng/mL. The calibrator was diluted with SDs. One hundred microliters of appropriate dilutions of the samples and calibrator were added to each well, and the plates were incubated at room temperature (22 ± 2°C) for 60 min. The plates were subsequently washed five times with TBST. The polyclonal rabbit anti-horse IgA H&L antibody conjugated with HRPO (Abcam, ab112871) was diluted 1:60 000 (100 µL per well), and the conjugate was diluted in TBST. The plates were incubated at room temperature (22 ± 2°C) in the dark for 50 min. The color reaction was developed with a supersensitive TMB substrate (Sigma−Aldrich; 100 μL per well) in the dark at room temperature for 10 min. The reaction was stopped with 2 M H2SO4 (P.P.H. Stanlab Sp.J., Lublin, Poland; 100 μL per well). Optical density was measured at a wavelength of 450 nm via a μQuantum™ ELISA microplate reader (BioTek Instruments). The IgA concentration was calculated via BioTek Gen5 Microplate Data Analysis Software. All samples and calibrators were assayed in duplicate. The intra-assay CV for IgA concentrations in fecal samples was 3.5%, and the interassay CV was 3.8%.

#### 2.2.4 Fecal microbiome analysis

The microbiome analysis of feces was conducted via *16S rRNA* gene amplicon sequencing. Microbial DNA from the stool samples was extracted via a standard procedure with the QIAamp PowerFecal Pro DNA Kit (Qiagen). To prepare DNA libraries for 16S rDNA amplicon sequencing, the QIAseq 16S/ITS panel (Qiagen), which targets the variable regions V3-V4 and V7-V9 of the *16S rRNA* gene, was used. The 16S rDNA amplicons were sequenced on a MiSeq platform (Illumina) via paired-end sequencing (2 × 276 bp) with the v3 MiSeq Reagent Kit (600 cycles). Data preprocessing was performed via QIIME2 with additional plugins (17). A custom script that utilizes *cutadapt* was employed to trim the V3–V4 and V7–V9 phased primers and perform demultiplexing (18). A quality check of the sequencing reads was performed with the *summary* method from the *demux* plugin. The dada2 plugin for paired-end reads was used for trimming, denoising, dereplication and chimera filtering (19). We used *sidle*, a Qiime2 plugin that implements the SMURF algorithm to analyze microbiomes on the basis of variable regions V3-V4 and V7-V9 (20). The SILVA 128 database was used as a reference, as it is compatible with *sidle* (21, 22). The subsequent bioinformatics and statistical analyses were performed in Python, as detailed below.

#### 2.2.5 Analysis of short-chain fatty acids

Analysis of SCFAs in the stool extracts was performed as previously described with modofication (23). Briefly, stool samples were extracted with 70% ethanol. The debris was removed via centrifugation, and 500 μL of the supernatant was transferred to a new tube and mixed with 50 μL of internal standard (2-ethylbutyric acid, 200 mM in 50% aqueous methanol), 300 μL of dehydrated pyridine 3% v/v (Merck) in ethanol, 300 μL of 250 mM N-(3-dimethylaminopropyl)-N′-ethylcarbodiimide hydrochloride (Sigma−Aldrich) in ethanol, and 300 μL of 20 mM 2-nitrophenylhydrazine hydrochloride (Sigma−Aldrich) in ethanol. The samples were incubated at 60°C for 20 min and mixed with 200 μL of sodium hydroxide solution (15% w/v with water and a sodium hydroxide solution/methanol ratio of 80/20, v/v) to terminate the reaction. After cooling, the mixture was extracted with 2 ml of phosphoric acid aqueous solution (0.5 mol/L) and 4 ml of diethyl ether twice; thereafter, the organic phase was collected and extracted with water. The upper phase containing the fatty acid derivatives was collected, evaporated, dissolved in 150 μL of methanol, and subjected to HPLC analysis. HPLC was performed via a 1525 Binary HPLC Pump with a 2489 UV/Visible (UV/Vis) detector (Waters) and a C18 column (Shimadzu 5 µm C18-120 Å, 250x4.6 mm), with a mobile phase of acetonitrile-methanol-water (30:16:54). The column temperature was 50°C, the flow rate was 1 mL/min, and the wavelength was 400 nm (23, 24).

#### 2.2.6 Statistical analysis

All the statistical analyses in this study were performed via Python. The libraries Pandas (25, 26), Matplotlib (27, 28), Numpy (29), Seaborn (30), and Skbio (31) were employed for computations and generating plots. The differences in alpha diversity between the probiotic and control groups were assessed via boxplots for four metrics across five time points, and statistical comparisons were made via the Mann−Whitney test to compare the alpha diversity metrics between these two groups at each time point and Wilcoxon tests between consecutive time points within the groups. Volatility plots were used to visualize the alpha diversity metrics over time, displaying individual data, group means, and standard deviation bars, with control limits set at ±2 and ±3 standard deviations from the global mean. The p values from the statistical tests were corrected for multiple testing via the Benjamini−Hochberg procedure. Generalized linear mixed effects (GLME) models were fitted for each alpha diversity metric to analyze changes over time and between groups. The models included time, group, and their interaction as fixed effects, with individual horses as a random effect. To assess beta diversity in the study groups, Weighted UniFrac was used, and the results were visualized through principal coordinate analysis (PCoA). Differences between groups were evaluated via PERMANOVA with 9999 permutations. Volatility plots were generated to visualize the Weighted UniFrac distance to the previous time point and the distance between the baseline (D0) and each subsequent time point (D28, D56, D84, and DX for the control and probiotic groups). Heatmaps were generated to illustrate the beta diversity metric distances between all samples in the study. To investigate whether there were significant differences in SCFA concentrations between the groups over time, we presented the obtained measurements in box plots and performed statistical tests at each time point: the Mann−Whitney test for comparisons between groups and the Wilcoxon test for comparisons within each group across different time points. For acetic acid and propionic acid, the most abundant SCFAs, a linear increasing trend over time was observed. Therefore, an GLME model was applied to determine if there was indeed an increase in the concentrations of acetic acid and propionic acid over time. Similar to other cases, to test whether there were differences between the groups in the context of IgA and EPG, the Mann−Whitney test was applied between the groups, and the Wilcoxon test was used within each group at each time point with Benjamini−Hochberg correction. To identify which bacterial taxa undergo changes over time and whether these changes are specific to the test group treated with probiotics versus the control group, we used the Analysis of Compositions of Microbiomes with Bias Correction 2 (ANCOM-BC2) in R. In this analysis, we accounted for fixed effects by including both time and group. Random effects were included to account for individual variability over time, ensuring that the model accurately reflected the natural differences among individual subjects.

## 3 Results

Over the course of the experiment, no episodes suggestive of gastrointestinal disease occurred in the test horses, and there was no change in fecal frequency or differences in fecal structure. The owner did not notice any negative effects in either group.

### 3.1 Microbiome alpha diversity

**Figure 1:**
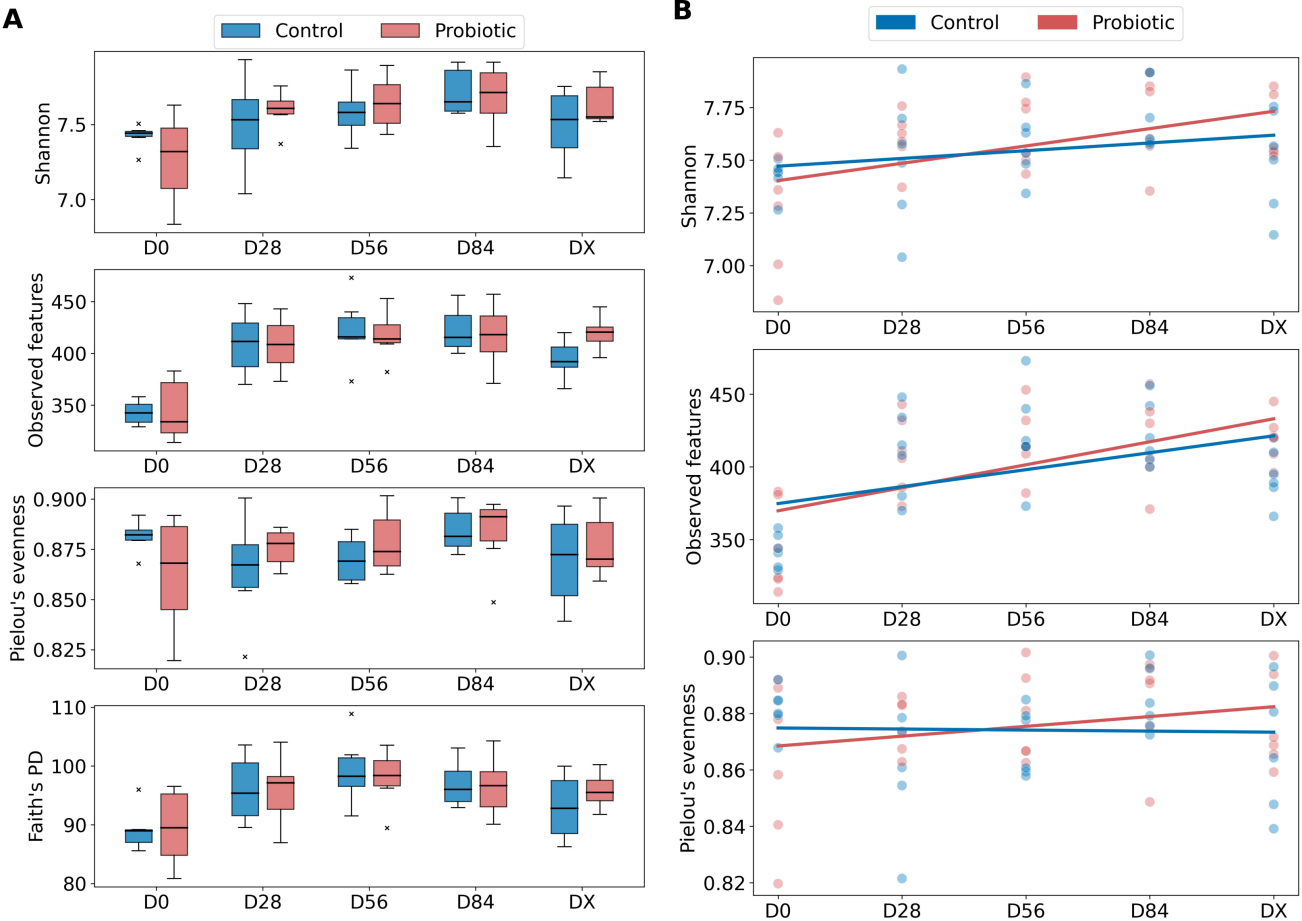
Differences in alpha diversity between the probiotic and control groups. **(A)** Boxplots for four alpha diversity metrics: Shannon entropy, observed features, Pielou’s evenness, and Faith’s PD at five different time points (D0, D28, D56, D84, DX); 25th percentile (Q1) as the bottom of a box, Q2 as the median, and 75th percentile (Q3) as the top of a box. The outliers are marked with an “x” (control group - blue, probiotic group - red color). **(B)** Generalized linear mixed model used to analyze the changes in alpha diversity (Shannon, observed features, Pielou’s evenness) over time (D0, D28, D56, D84, DX) and between groups (control group - blue, probiotic group - red color).

Four alpha diversity metrics were analyzed: Shannon entropy, observed features, Pielou’s evenness, and Faith’s PD at five different time points (D0, D28, D56, D84, DX) (**Figure 1A**). The Mann−Whitney test was performed to compare the alpha diversity metrics between these two groups at each time point. No statistically significant difference was detected, indicating that the use of the probiotic did not have an effect on alpha diversity. The results of the Mann−Whitney and Wilcoxon tests are summarized in **Supplementary Tables 1**, **2**. To test whether statistically significant differences occurred between consecutive time points within the groups, we performed the Wilcoxon test for dependent samples. However, no statistically significant differences were detected.

To further analyze the changes in alpha diversity over time and between groups, GLME models were fitted for each alpha diversity metric (**Figure 1B**). Notably, this model was not successfully fitted with Faith’s PD metric because, as shown in the plots (**Figure 1A**), this alpha diversity metric represents a nonlinear trend over time, with an increase from D0 to D56, followed by a decrease to DX. The baseline (D0) expected Shannon entropy was 7.435 for the control group (except for 7.435, se = 0.093, P < 0.001). The coefficient for the probiotic group (−0.114, ± 0.132, P = 0.387) indicates no significant difference in Shannon entropy between the probiotic and control groups at the initial time point.

A positive trend in Shannon entropy over time may be indicated by the time coefficient (0.037, se = 0.027, P = 0.167), but this effect is also not statistically significant. The interaction term of time and group (0.045, se = 0.038, P = 0.226) suggested a slightly greater increase in Shannon entropy over time in the probiotic group than in the control group; however, this small effect was also not statistically significant. The variance of the random effect for individual horses is 0.005 (se = 0.033), indicating minimal variability in Shannon entropy attributable to differences between horses.

The expected number of observed features in the D0 for the control group is 363.150 (intercept 363.150, se = 13.965, P < 0.001). The coefficient for the probiotic group (−9.133, se = 19.750, P = 0.644) suggests a lower number of observed features in the probiotic group compared to the control group at the initial time point, which is before probiotic administration, although this difference is not statistically significant. The time coefficient indicates a significant positive trend in the number of observed features over time (increase by 11.650, se = 4.210 for each successive time point, P = 0.006). The interaction term (time and group, 4.167, se = 5.954, P = 0.484) suggests a slightly higher increase in the number of observed features over time in the probiotic group compared to the control group, However, this effect is not statistically significant. The variance of the random effect for individual horses is 0.236 (se = 5.061), indicating minimal variability in the number of observed features attributable to differences between horses.

The expected Pielou’s evenness for the control group at the baseline (D0) is 0.875 (se = 0.008, P < 0.001). The coefficient for the probiotic group (−0.010, se = 0.011, P = 0.363) suggests a slightly lower Pielou’s evenness in the probiotic group compared to the control group at the initial time point, although this difference is small and not statistically significant. There is no significant change in Pielou’s evenness over time (0, se = 0.002, P = 0.868). There is also no significant difference between groups, as the interaction term (time and group, 0.004, se = 0.003, P = 0.222) suggests marginal increase in Pielou’s evenness over time in the probiotic group compared to the control group, and this effect is not statistically significant. The variance of the random effect for individual horses is 0, se = 0.003, indicating minimal variability in Pielou’s evenness attributable to differences between horses.

### 3.2 Microbiome beta diversity

**Figure 2:**
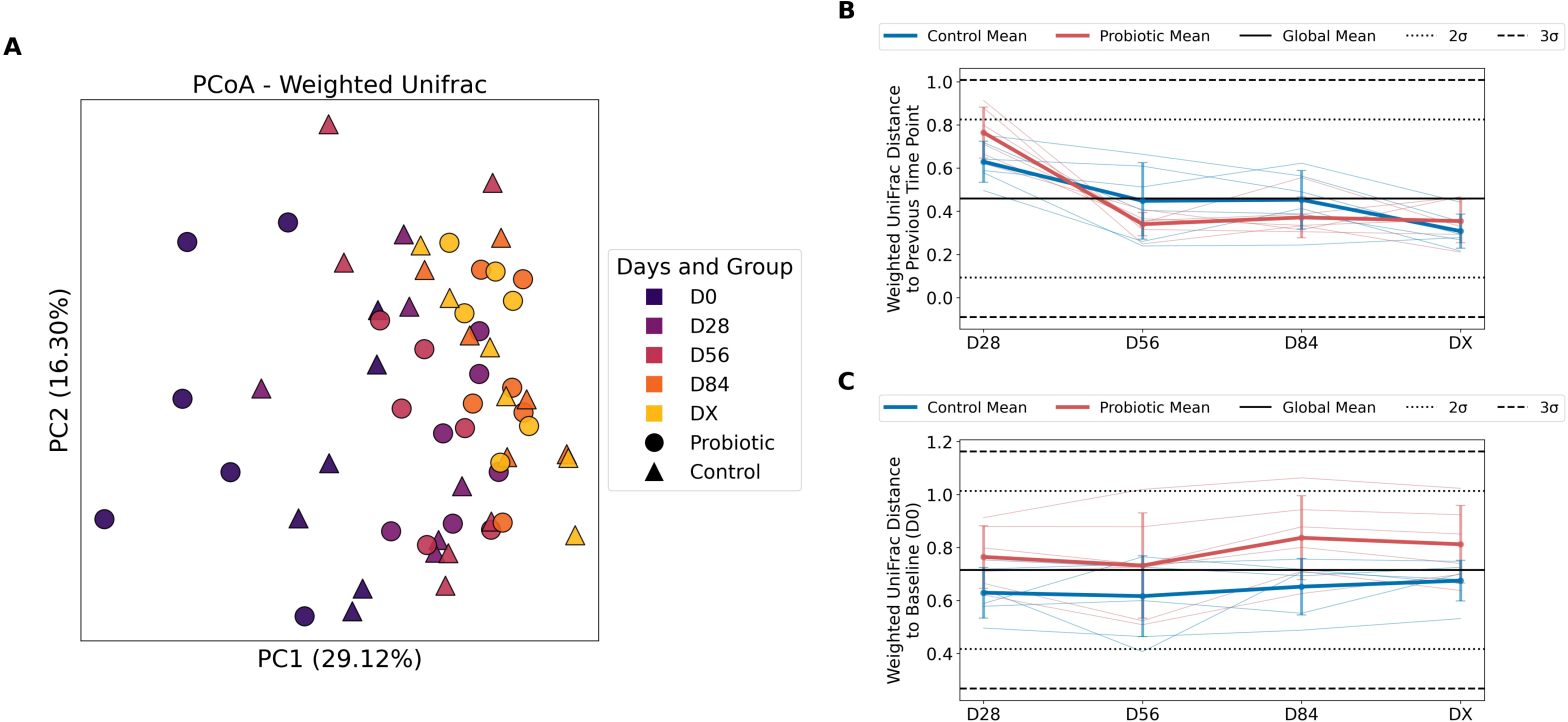
Differences in beta diversity between the probiotic and control groups (Weighted UniFrac). **(A)** Principal coordinate analysis (PCoA) for the Weighted UniFrac distance. The circles on the plot represent samples from the probiotic group, and the triangles represent controls. The colors correspond to different time points. **(B)** Weighted UniFrac distance to the previous time point for two groups: the control and probiotic groups. The blue lines represent the mean Weighted UniFrac distances for the control group, with individual data points shown by thin lines. Red lines represent the mean Weighted UniFrac distances for the probiotic group, with individual data points shown in lighter red. The individual data points are shown by thin lines in corresponding colors. The solid black line indicates the global mean Weighted UniFrac distance. The dotted black lines represent ±2 standard deviations (± 2σ) from the global mean. The dashed black lines represent ±3 standard deviations (± 3σ) from the global mean. The error bars indicate the standard deviation for the respective groups at each time point. The length of the error bars shows the variability in Weighted UniFrac distances within each group. The values at D28 represent changes between the initial point (D0, not shown on this plot) and the time points D28, D56, D84, and DX, which represent changes from the previous time point to the current time point (e.g., D56 shows changes from D28 to D56). The y-axis shows the Weighted UniFrac distance, which quantifies the difference in community composition that takes into account phylogenetic distance and the abundance of features between successive time points for the same individual; **(C)** Weighted UniFrac distance between the baseline (D0) and each subsequent time point (D28, D56, D84, and DX).

To assess beta diversity in the study groups, Weighted Unifrac was used. The PCoA for the Weighted UniFrac, which provides a nuanced view by incorporating both the evolutionary distances and the abundance of taxa, making it sensitive to changes in both the presence/absence and the abundance of features, is visualized in **Figure 2A**. The first principal coordinate (PC1), which represents the direction in the data that explains the most variation among the samples, accounts for 29.12% of the variation. At the initial time point (D0) before administration of the probiotics, we observed statistically significant differences between the groups (P = 0.0124); however, after multiple comparisons were corrected (P adjusted = 0.062), these differences were no longer significant. At later time points, the differences between the control and probiotic groups were not statistically significant (**Supplementary Table 6**).

To illustrate the changes in the microbial community structure over time, the data are presented in volatility plots, showing the Weighted UniFrac distance to the previous time point (**Figure 2B**). The values at D28 reflect the changes in community composition from the baseline (D0) to the first measured time point (D28). Both groups presented relatively high Weighted UniFrac distances at D28, indicating substantial changes in community composition during the first 28 days of the study. From D28 to D56, both groups showed a significant decrease in Weighted UniFrac distance to the previous time point, suggesting that the community composition became more stable after the initial 28 days, which corresponds to the first 28 days of probiotic administration to the experimental group. Compared with the probiotic group, the control group presented greater variance in D56. Within the Probiotic group, the decrease in Weighted UniFrac distance during this period was greater, indicating a minor change to the previous time point within the Probiotic group compared with the Control group. The Weighted UniFrac distances in both groups stabilized further (D84), oscillating around similar values (which indicates a similar pace of change over time compared with the previous time point). In DX, the control group showed less change than did the probiotic group.

**Figure 2C** presents the Weighted UniFrac distance to baseline. The distances from the baseline (D0) remained relatively constant over time for both groups, with the exception of D84 in the Probiotic group. The time point D84 in the Probiotic group was the greatest distance from the baseline (D0). This consistency suggests that the community composition changes introduced by the initial intervention are maintained throughout the subsequent time points. Compared with the control group, the probiotic group consistently presented greater distances at all time points. These findings indicate that the community composition in the Probiotic group deviated more from the baseline than did that in the Control group. The error bars suggest great variance in the community composition among individuals in both groups.

The heatmap in **Supplementary Figure 1** illustrates the distances in the metric between all samples in the study. The samples are arranged individually, where C1 denotes horse 1 from the control group, P1 denotes individual 1 from the probiotic group, and so on. For each individual, the subsequent time points are displayed. Individuals at all their time points are highlighted with a red frame. **Supplementary Figure 1A** presents this heatmap but is divided into groups, where each individual across all time points is highlighted with a black frame. The heatmap in **Supplementary Figure 1B** (and divided by groups in **Supplementary Figure 1C**) shows the same data but arranged by time point and then by individual. The red frame in **Supplementary Figure 1A** encompasses a single time point and all individuals. From these visualizations illustrating distances in the beta diversity metric, it can be observed that the greatest differences (the lightest points on the heatmaps divided by groups and more yellow on the larger heatmaps, closer to a value of 1.0) occur between D0 and the subsequent time points, particularly in the probiotic group (**Supplementary Figure 1C**). This finding indicates that the most significant change in the microbiome compared with that at baseline occurred, and it was more prominent in the group in which the probiotic was administered. The passage of time had a substantial impact on both groups, and the use of probiotics as an additional differentiating factor between the groups cannot be excluded.

### 3.3 Microbiome taxonomy

#### 3.3.1 Abundance of probiotics used

**Figure 3:**
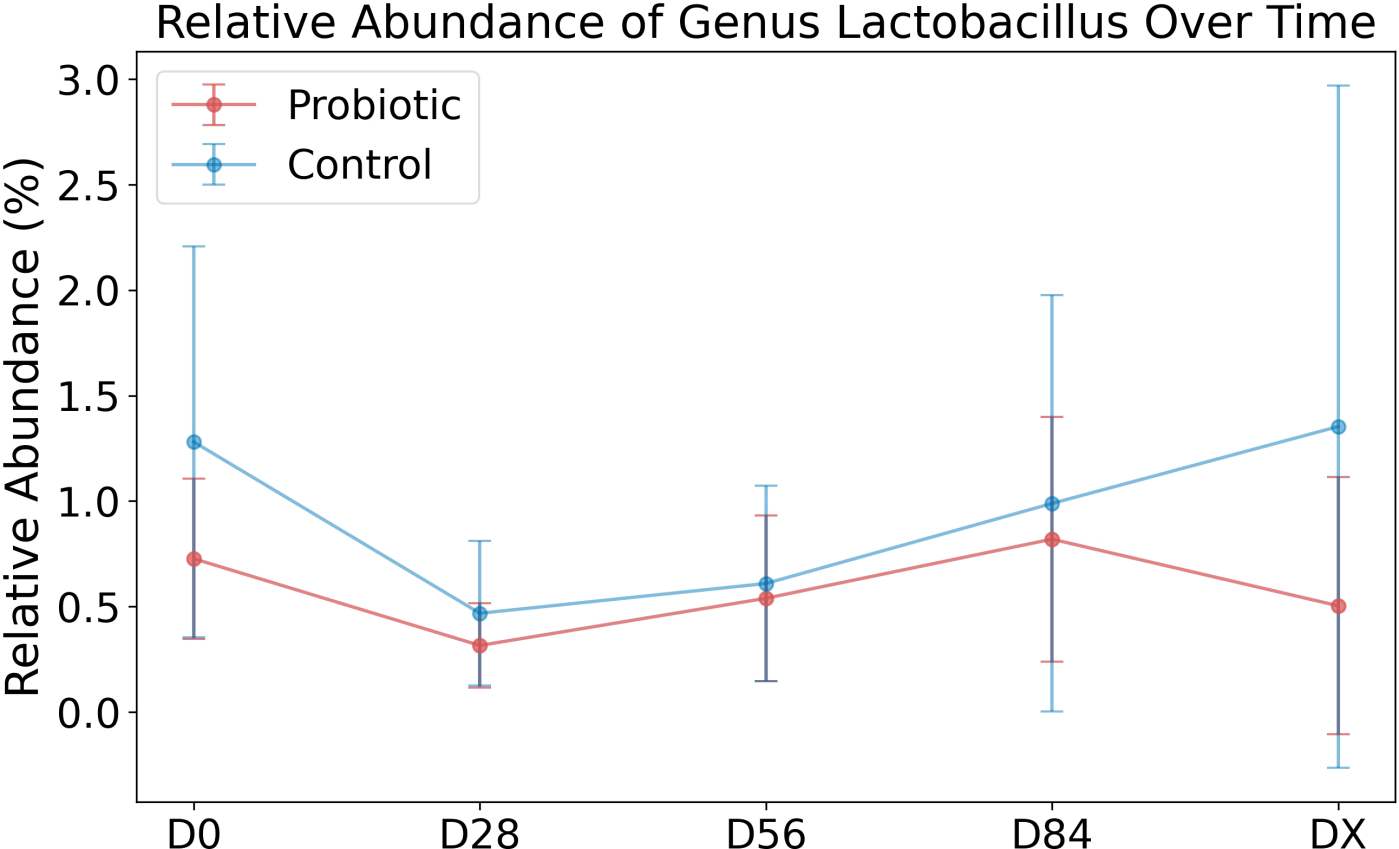
Relative abundance of the genus Lactobacillus over time. The relative abundance of Lactobacillus over time in two groups: the probiotic group (in red) and the control group (in blue). The points on the plot represent the mean relative abundance of this taxon at each time point (D0, D28, D56, D84, DX) for each group. The error bars around the points indicate the standard deviation, reflecting the variability within each group at the respective time points.

We wanted to determine whether our sequencing data reflected an increased abundance of the supplemented genera *Lactobacillus rhamnosus* NCIMB 8010 (family *Lactobacillaceae*, genus *Lactobacillus*), *Pedicoccus accidilactici* NCIMB 8018 (family *Lactobacillaceae*, genus *Pediococcus*), and *Enterococcus faecium* NCIMB 11181 (family *Enterococcaceae*, genus *Enterococcus*) in the probiotic group. As *16S rDNA* sequencing allows for identification at the genus level (32), *Lactobacillus* was identified, but *Pediococcus* was not present in our dataset, indicating that this genus was not detected. The mean relative abundances of *Lactobacillus* in the groups are shown in **Figure 3**. In the control group, the average abundance of the genus *Lactobacillus* was greater than that in the probiotic group. Conversely, *Enterococcus* was practically absent in the data, with two exceptions: its presence was identified in only 2 samples from the control group, one sample (C5–D56, 0.037%) and another sample (C4–DX, 0.011%).

#### 3.3.2 Differential abundance

**Figure 4:**
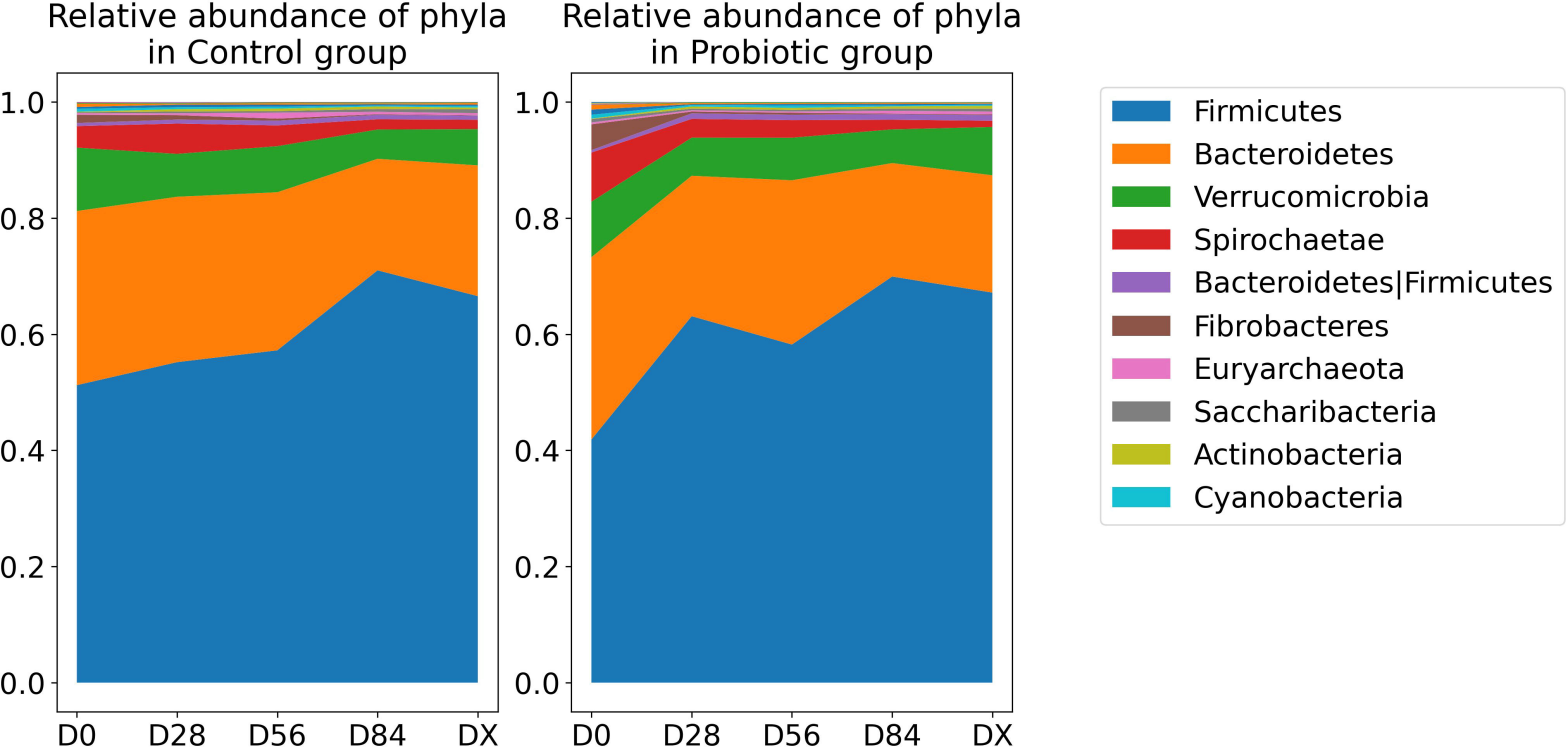
Relative abundance of the dominant phyla over time. The legend lists the top 10 most abundant phyla in descending order of abundance.

**Figure 5:**
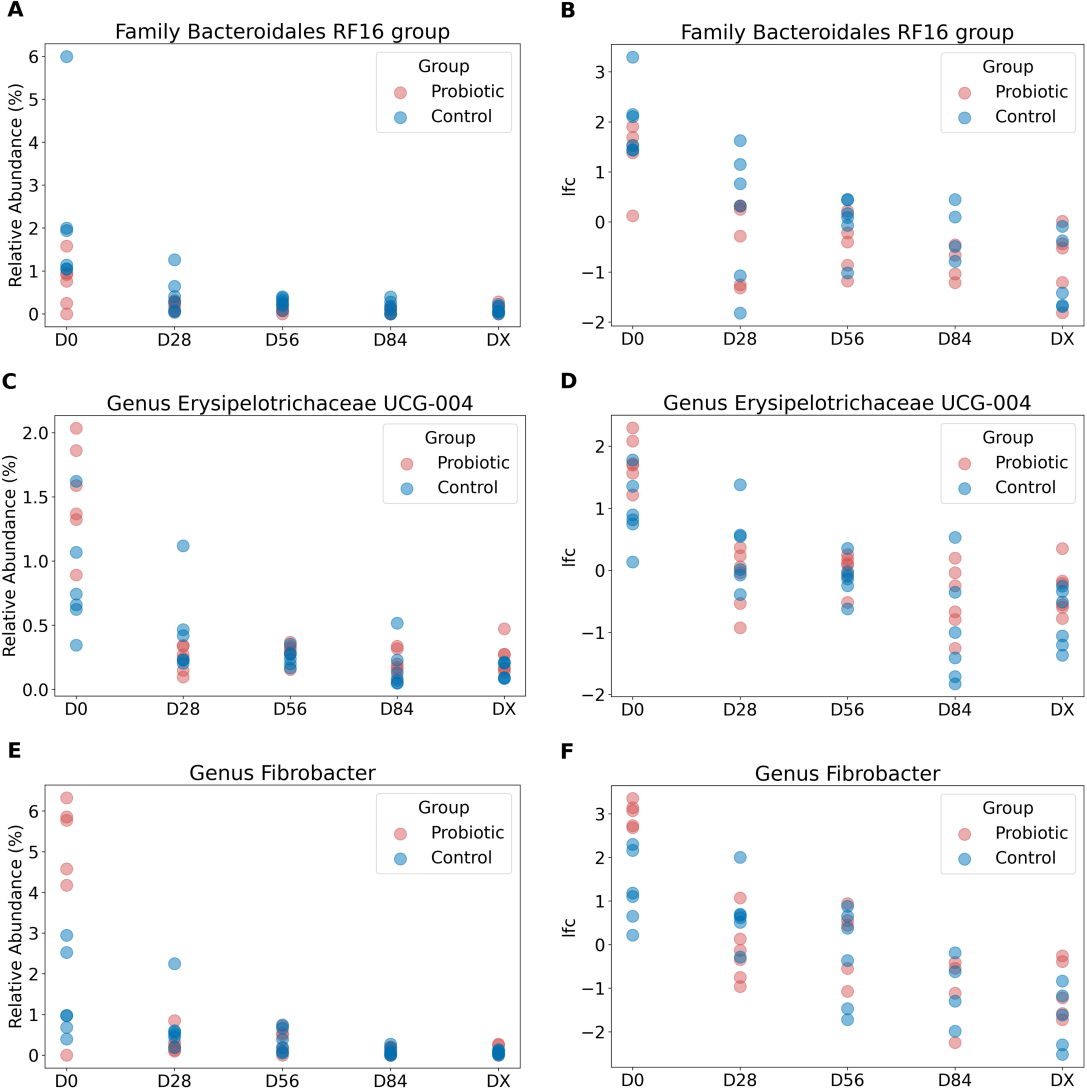
The relative abundances of taxa identified as significantly changing over time. The relative abundances of taxa identified as significantly changing over time—the family Bacteroidales RF16 group, genus Erysipelotrichaceae UCG-004, and genus Fibrobacter—are shown in **(A, C, E)**. **(B, D, F)** display clr-transformed and bias-corrected values of these taxa from the feature table generated by ANCOM-BC2. A significant decrease in the abundance of these taxa over time was detected across all groups.

In **Figure 4**, the relative abundances of taxa at the phylum level over time are shown. To identify which bacterial taxa undergo changes over time and whether these changes are specific to the test group treated with probiotics versus the control group, we employed ANCOM-BC2 (32). Significant changes were detected in three taxa: the family *Bacteroidales* RF16 group, the genus *Erysipelotrichaceae UCG-004*, and the genus *Fibrobacter*. In all the cases, there was a gradual decrease in abundance over time. The reported q values are p values adjusted via the Holm method. For the taxon identified as the family *Bacteroidales* RF16 group, the log fold change at the initial time point, lfc (intercept), at D0 was 2.13 (q = 0.002). There was a significant negative change over time, with a lfc of −0.656 per unit time (q = 0.003). However, there was no significant contribution from the probiotic group factor (lfc group probiotic = −0.987, but q = 1 and lfc time:group probiotic = 0.194, q = 1), indicating that the decrease was similar in both groups. All values passed the sensitivity analysis, indicating that they are robust to the addition of pseudocounts required for log transformation. The results are presented in **Figure 5B**. Additionally, **Figure 5A** shows the relative abundance (before bias correction). For *Erysipelotrichaceae UCG-004*, the log fold change at the initial time point, lfc (intercept) at D0, was 1.32, however this is not significant (q = 0.091), and sensitive to pseudocount addition. This means that it cannot be concluded that the intercept in the model is different from 0. Nevertheless, there was a significant negative change over time, with a lfc of −0.479 per unit time (q = 0.009). Similarly, in this case there was no significant contribution from the probiotic group factor (lfc group probiotic = 0.199, but q = 1 and lfc time:group probiotic = 0.028, q = 1), indicating that the decrease was similar in both groups. The results are presented in **Figure 5D**. Additionally, **Figure 5C** shows the relative abundance (before bias correction). For the *Fibrobacter*, the log fold change at the initial time point, lfc (intercept) at D0, was 2.1, which is significantly different from 0 (q = 0.044). There was a significant negative change over time, with a lfc of −0.772 per unit time (q = 0.0008). However, similarly to *Bacteroidales* RF16 group and *Erysipelotrichaceae UCG-004*, there was no significant contribution from the probiotic group factor (lfc group probiotic = 0.615, but q = 1 and lfc time:group probiotic = −0.097, q = 1), indicating that the decrease was similar in both groups. All values passed the sensitivity analysis. The results are presented in **Figure 5F**. Additionally, **Figure 5E** shows the relative abundance (before bias correction).

### 3.4 SCFAs

**Figure 6:**
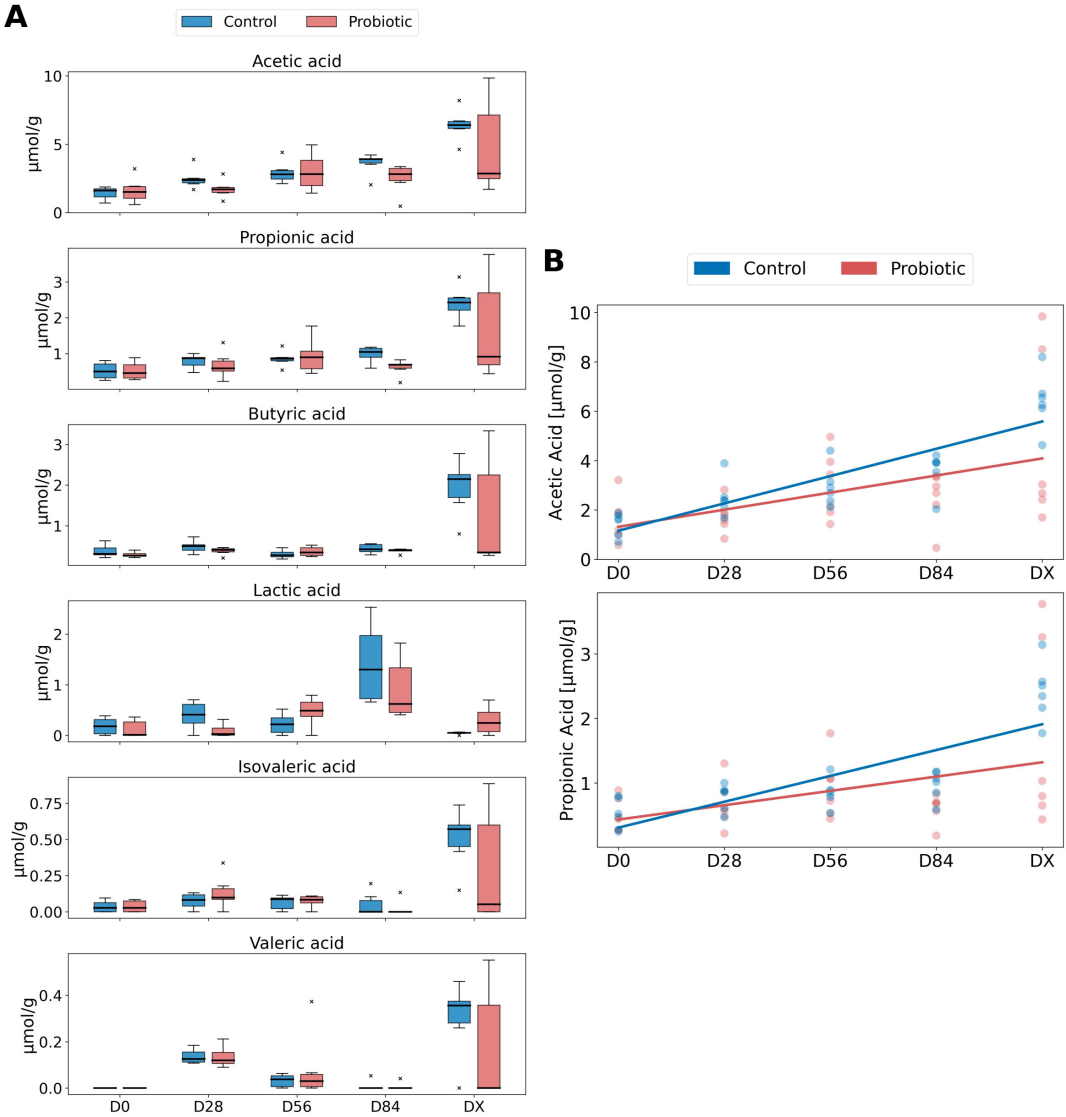
SCFA concentrations between the groups over time. **(A)** Box plots displaying the concentrations of SCFAs, acetic acid, propionic acid, butyric acid, lactic acid, isovaleric acid, and valeric acid, across different time points (D0, D28, D56, D84, DX) for both the control and probiotic groups. The box plots represent the interquartile range (IQR) with the 25th percentile (Q1) as the bottom of the box, the median (Q2) as the line within the box, and the 75th percentile (Q3) as the top of the box. The outliers are marked with an “x.” **(B)** Generalized inear mixed effects (GLME) model, illustrating the trends in acetic acid and propionic acid concentrations over time for the control (blue) and probiotic (red) groups. The lines depict the estimated values from the GLME model, with individual data points shown to represent the distribution within each group at different time points.

The SCFAs identified in the feces were acetic acid, propionic acid, butyric acid, lactic acid, valeric acid, and isovaleric acid (listed in decreasing order of abundance). The SCFA content results are shown in **Figure 6A**. To investigate whether there were significant differences in SCFA concentrations between the groups over time, we presented the obtained measurements in box plots (**Figure 6A**) and performed statistical tests at each time point both between the groups and within each group across different time points. For acetic acid and propionic acid, the most abundant SCFAs, a linear increasing trend over time was observed (**Figure 6B**). Therefore, we applied an GLME model to determine if there was indeed an increase in the concentrations of acetic acid and propionic acid over time.

The predicted value of acetic acid in the control group at the beginning of the study (D0) was 0.052 μmol/g. However, this result is not statistically significant (P = 0.934), indicating that we cannot confidently assert that this value differs from zero. Importantly, this is a model fitting result, and in reality, the average acetic acid concentration in the control group is different from zero. The average acetic acid level in the probiotic group was 0.572 higher than that in the control group, but this difference was not statistically significant (P = 0.518). Each subsequent time point is associated with a statistically significant increase in acetic acid levels of 1.106 μmol/g per time unit (P < 0.001). This suggests that, irrespective of the group, acetic acid levels increase over time. The interaction between time and the probiotic group indicates a reduction in the increase of acetic acid by 0.414 in the probiotic group (−0.414) compared to the control group, but this result is not statistically significant (P = 0.116). The variance between groups is 0.069, indicating minimal differentiation between the groups.

The mean value of propionic acid for the control group at the beginning of the study (D0) is −0.089 units, which is the best fit of the model. However, this result is not statistically significant (P = 0.736), indicating that we cannot confidently assert that the actual mean value differs from zero. The probiotic group has an average propionic acid level higher by 0.303 units compared to the control group, but this difference is not statistically significant (P = 0.415). Each subsequent time point is associated with a statistically significant increase in propionic acid levels by (0.400) units (P < 0.001). This suggests that, irrespective of the group, propionic acid levels increase over time. The interaction between time and the probiotic group indicates a reduction in the increase of propionic acid by 0.178 units in the probiotic group (−0.178) compared to the control group, but this result is not statistically significant (P = 0.112). The variance between groups is (< 0.001), suggesting no differentiation between the groups.

### 3.5 SIgA

**Figure 7:**
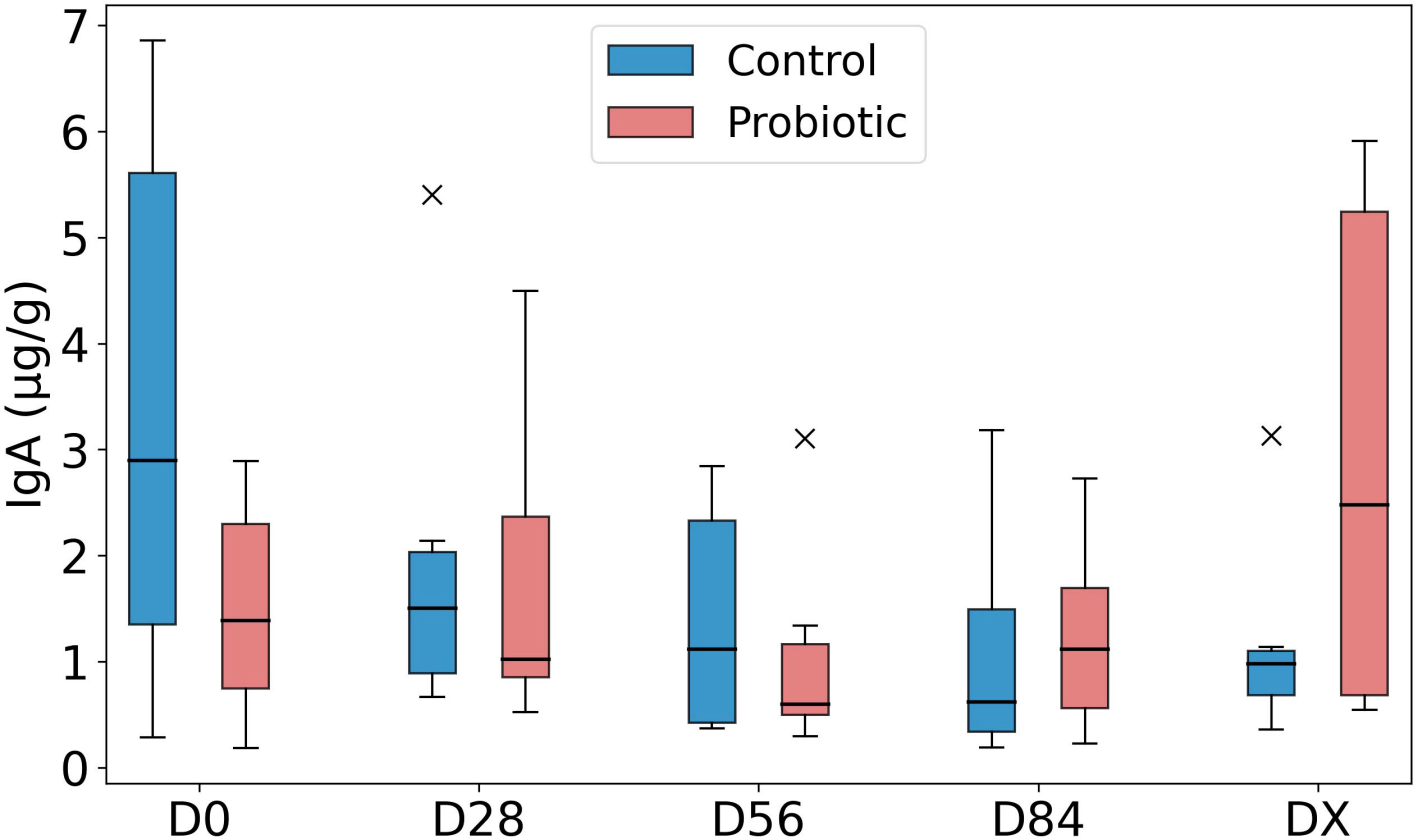
Fecal SIgA concentration. Legend: Fecal secretory IgA (SIgA) concentration expressed in µg/g of feces, presented as a box plot for the control group and the probiotic group. The bottom of the box represents the 25th percentile (Q1), the middle line represents the median (Q2), and the top of the box represents the 75th percentile (Q3). The outliers are marked with an “x”.

The SIgA content in feces, expressed in μg/g, is presented in **Figure 7**. As shown in the figure, the data dispersion within the groups was considerable, especially in the control group at D0 and the probiotic group at DX. No significant changes over time were detected, nor were there any statistically significant differences between the groups at any time point.

### 3.6 EPG

No significant differences were found between the groups at any time point, nor were there any differences within the groups over time. There was a lack of correlation between the EPG and fecal SIgA levels.

## 4 Discussion

The present study revealed no effect of long-term (84 days) use of a commercial probiotic dietary supplement on either mucosal immunity (level of fecal SIgA) or the composition of the intestinal microbiota, which has the ability to produce SCFAs, in healthy horses. However, in-depth observations suggest the most significant change in the microbiome compared with that at baseline, particularly in the group in which the probiotic was administered. While the passage of time had a substantial impact on both groups, the possibility that the probiotic serves as an additional differentiating factor cannot be excluded. Importantly, however, we do not have statistically significant evidence to support this difference.

Data available in the literature from mouse and human medicine do not indicate a clear effect of oral probiotics on fecal SIgA secretion. This may be due to the type of strains used in the supplement, colonization potential, composition and interaction of the supplement components with intestinal epithelial cells. Studies in mice have shown that peroral administration of *Bifidobacterium bifidum* enhances intestinal IgA production mainly by increasing the number of IgA-secreting cells (12). In children, the use of another strain, also *Bifidobacterium* (*B. lactis*), in a 4-week trial resulted in an increase in fecal SIgA secretion (11). Another study revealed no effect of a *Lactobacillus rhamnosus/helveticus* probiotic on stool IgA concentrations in children with gastroenteritis (13). A significant increase in SIgA and SCFA production was noted after 4 weeks of probiotic (*Lactobacillus acidophilus, Bifidobacterium lactis*) use in adults with diarrhea or constipation (14). In the present study, there were no differences between SIgA values in horses, either between groups or over time—all horses had results corresponding to the average result for healthy horses obtained in the authors’ previous studies (4 µg/g feces) (3). However, the correlations between probiotics and SIgA secretion were positive when strains from *Bifidobacterium* spp., which were not used in the preparation in the present study, were used. This may suggest the need for a larger study including other probiotic strains dedicated to horses.

The results of the present study revealed no significant differences in microbiome alpha diversity, except for an increase in the number of observed features over time, regardless of the group, which was detected by the GLME model. The equine stool microbiome remains stable over time, and there is no evidence of an impact from the use of probiotics. The greatest differences (microbiome shifts, measured in terms of distances) can be observed between the baseline (D0) and D28, which corresponds to the starting point before the probiotic was administered to one group and after 28 days of its use. This is what was expected: after probiotic administration, the microbiome would shift. However, unfortunately, the probiotic and control groups significantly differed at D0, which was not expected, as the study groups were thought to be similar in terms of the microbiome at baseline. As these differences seem to be stronger in the group that received the probiotic, the influence of the probiotic on the microbiome in the experimental group cannot be dismissed, despite the unfortunate difference observed at D0. Despite the equal and randomized selection of horses into both groups among healthy horses maintained under identical conditions, the error of significant differences in microbiome composition between horses in the two groups at D0 could not be avoided. This error was not eliminated at the beginning of the study because the samples were subjected to pooled analysis after the study trial. Little is known about how probiotic use affects the composition of the microbiome in horses. A single *in vivo* study revealed a positive effect of probiotic use on the composition of the microbiome in foals (6), but only a trend was observed for prebiotic effects on the diversity indices of the GI microbiome in adult stallions (p= 0.07) (7). *In vitro* studies conducted in cultures of equine intestinal contents revealed no effect of probiotic application at 24 or 48 hours on alpha or beta diversity indices and noted limited taxonomic differences (8). However, differences in the production of SCFAs, such as acetate and propionate, have been noted (8). The lack of differences in microbiome parameters associated with the use of probiotics in the study presented here and in the study by MacNicoll et al., 2023 may be primarily due to their bioavailability as well as their ability to affect the horse’s mature microbiota. Significant changes were only seen in foals in two independent studies (6, 33). In the present study, we observed a decrease in the relative frequency of three taxa during experiment: the family *Bacteroidales* RF16 group, the genus *Erysipelotrichaceae* UCG-004, and the genus *Fibrobacter*. The probiotic did not cause changes in the most important phyla for horse GI, i.e., *Firmicutes and Bacteroidetes.* However, significant differences were detected in the genus *Fibrobacter* (an important part of the phylum *Fibrobacteres*), namely, a decrease over time in the both test groups. The literature indicates a decrease in the abundance of this bacterium in the course of metabolic disorders/obesity in horses; hence, more cautious use of probiotics in this group of horses may be necessary in the future because of the risk of enhancing the effect (34). On the other hand, the genus *Erysipelotrichaceae*, whose abundance decreased in the both study groups, increased in correlation with the body condition score in obese horses, according to the literature (34). The horses used in the study were in normal condition and presented no signs of metabolic disorders on clinical examination. *Bacteroidales* RF116 is described in horses and, according to Edwards et al., 2020, belongs to the core taxa; however, its contribution has not been described in the literature (35). The differences noted do not seem to have a significant effect on the homeostasis of the microbiome in the horses studied. These changes showed a time dependence greater than that of the groups tested, which may be related to a temporal change in pasture composition (plant growth at the beginning of spring) or a change in the composition of hay from a commercial source.

We wanted to determine whether our sequencing data reflected an increased abundance of the supplemented genera *Lactobacillus rhamnosus* NCIMB 8010 (family *Lactobacillaceae*, genus *Lactobacillus*), *Pedicoccus accidilactic*i NCIMB 8018 (family *Lactobacillaceae*, genus *Pedicoccus*), and *Enterococcus faecium* NCIMB 11181 (family *Enterococcaceae*, genus *Enterococcus*) in the probiotic group. Among those listed, only the genus Lactobacillus was identified, while there was no effect of long-term probiotic use on the relative abundance of the supplemented genera. One limitation of the present study was the lack of fecal examination at 24 and 48 hours after the start of administration, which made it impossible to assess the growth in the gastrointestinal tract of the bacterial strains used in the formulation (which could be compared with the *in vitro* results of MacNicoll et al.) (8). However, the lack of isotonic changes in the composition of the microbiome, as well as the detectability of the applied strains in the feces after 28 days of continuous administration, indicates both a lack of bacterial growth and a lack of colonization of the gastrointestinal tract in the tested horses. According to the data of Schoster et al. (2014), this could be due to several factors, such as a lack of strains or adequate amounts of strains in the formulation or a mismatch in the selection of strains by species (1). Research conducted by Yuki et al., 2000 demonstrated that *Lactobacillus* strains isolated from horse feces adhered to epithelial cells in laboratory conditions, indicating their potential to colonize the mucosa (36). This implies that there should have been a rise in the relative occurrence of *Lactobacillus* spp. in the horse feces of the study group included in the current research. Conversely, an *in vivo* study by Yuyama et al. that involved administering a probiotic to foals, comprising strains originally sourced from horse feces (*L. salivarius*, *L. reuteri, L. crispatus*, *L. johnsonii*, and *L. equi*), has demonstrated gastrointestinal colonization and detection of the strains in the feces (33). The source of the *Lactobacillus rhamnosus* strains found in the formula remains unidentified. The decreased relative occurrence of Lactobacillus genus in the probiotic group could be attributed to the competitive impact of lactic acid bacteria from the formula on those originally present in the gastrointestinal tract. At the same time, the authors’ own research and that of Costa et al., 2015 showed that under physiological conditions, *Lactobacillus* and *Enterococcus* predominate in the small intestine and their relative abundance is not always reflected in the faeces (37, 38). Additionally, the potential for colonization may be heavily affected by the technical instability of the bacterial strain or the actual composition and bioavailability of the product. Weese 2002 noted that only 2/13 (15%) of veterinary and human probiotics contained the specified organism at the concentration indicated on the label, and all veterinary products contained <2% of the stated concentration (39). In the work presented here, the preparation used was not cultured to confirm its composition, relying on information obtained from the package insert and the manufacturer’s direct statement. Studies in the literature indicate that when probiotic strains are used, both foals and adult horses fail to achieve sustained colonization of the digestive tract (after 5 and 10 days of use, respectively) (1, 40). However, the strains were detected in the feces during probiotic application and several days later, which was not observed in the present study. This finding indicates that any beneficial effects of probiotics may not last beyond the period of administration, necessitating prolonged or repeated treatment; however, in the present study, no significant effects were observed despite prolonged administration (84 days).

Analysis via a GLME model for acetic and propionic acids revealed an increase in the production of these acids over time, which was noticeable in both groups. This may be related to seasonal changes in hay/vegetation composition in the pasture, which affected both groups in the same way, regardless of probiotic use. The recorded concentrations of acetate and propionate were astonishingly lower than those documented in existing literature on a μmol/g basis (23, 41). This significant disparity could be attributed to the diverse methodologies employed in the analysis processes, whether it be the nuanced techniques of gas-liquid chromatography or the sophisticated intricacies of high-performance liquid chromatography, as well as the specific manner in which the data were meticulously converted. Remarkably, in terms of percentage, acetate represented a dominant 59% and propionate a notable 24% of the total short-chain fatty acids, a distribution that resonates with findings observed in equines indulging in a diet predominantly composed of hay (41).

In addition to the already identified shortfall of not assessing the microbiome in crucial samples taken at 24 and 48 hours, the study faces significant challenges due to the limited sample size and the inherent potential for pronounced individual variations in microbiota responses. Despite meticulous efforts to standardize the maintenance conditions of the horses across both groups, the looming specter of diverse dietary or environmental influences on the observed results must also be prominently highlighted and thoroughly considered.

These data call into question the need for prophylactic probiotic use in horses during a change in diet or pasture. When creating the study plan, the authors did their best to limit the differences in the horses’ maintenance and feeding regimens, even taking into account how the probiotic was administered in carrots and its introduction into the diet in both groups, but the impact of seasonal changes could not be avoided.

## Data Availability

The datasets presented in this study can be found in online repositories. The names of the repository/repositories and accession number(s) can be found below: PRJNA1150766 (SRA).
