# Supplementary figures and images for: Investigating the potential immunomodulatory effects of commercial oral probiotic supplements on equine gastrointestinal tract barrier function
Source: Front Immunol. 2025 Jan 21;15:1487664. doi: 10.3389/fimmu.2024.1487664 (PMC11790434; doi:10.3389/fimmu.2024.1487664)

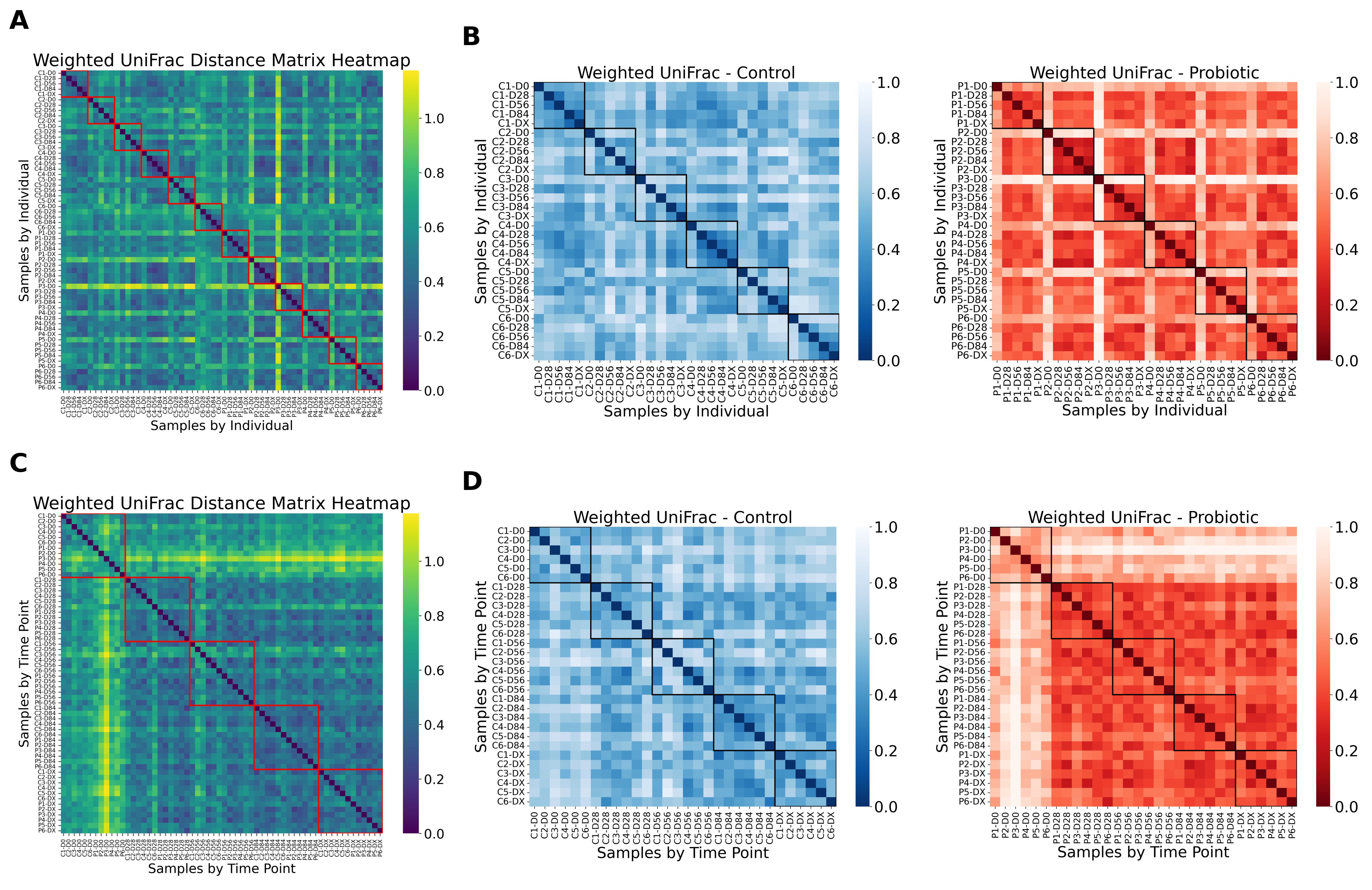

Supplement: Supplementary file 1 [file Image1.jpeg]
