# Supplementary material for: Investigating the potential immunomodulatory effects of commercial oral probiotic supplements on equine gastrointestinal tract barrier function
Source: Front Immunol. 2025 Jan 21;15:1487664. doi: 10.3389/fimmu.2024.1487664 (PMC11790434; doi:10.3389/fimmu.2024.1487664)
Supplement: Supplementary file 2 [file Table1.docx]

Supplementary Material

**Table 1:** Mann-Whitney test results for Probiotic vs. Control in each alpha metric for every time point. Corrected p-values were obtained using the Benjamini-Hochberg method.

| **Metric** | **Time Point** | **U Statistic** | **p-value** | **corrected**  **p-value** |
| --- | --- | --- | --- | --- |
| **Shannon** | D0 | 14.000 | 0.288 | 0.468 |
|  | D28 | 14.000 | 0.288 | 0.468 |
|  | D56 | 14.000 | 0.288 | 0.468 |
|  | D84 | 17.000 | 0.468 | 0.468 |
|  | DX | 12.000 | 0.189 | 0.468 |
| **Observed features** | D0 | 15.500 | 0.374 | 0.468 |
|  | D28 | 17.000 | 0.468 | 0.468 |
|  | D56 | 15.000 | 0.342 | 0.468 |
|  | D84 | 16.500 | 0.436 | 0.468 |
|  | DX | 4.500 | 0.019 | 0.370 |
| **Pielou’s evenness** | D0 | 11.000 | 0.149 | 0.468 |
|  | D28 | 12.000 | 0.189 | 0.468 |
|  | D56 | 10.000 | 0.115 | 0.468 |
|  | D84 | 18.000 | 0.468 | 0.468 |
|  | DX | 14.000 | 0.288 | 0.468 |
| **Faith’s PD** | D0 | 17.000 | 0.468 | 0.468 |
|  | D28 | 16.000 | 0.405 | 0.468 |
|  | D56 | 16.000 | 0.405 | 0.468 |
|  | D84 | 16.000 | 0.405 | 0.468 |
|  | DX | 13.000 | 0.236 | 0.468 |

**Table 2:** Wilcoxon test results for alpha diversity metrics comparing successive time points within each group (Control and Probiotic). Corrected p-values were obtained using the Benjamini-Hochberg method.

| **Metric** | **Group** | **Time Point 1** | **Time Point 2** | **Statistic** | **p-value** | **corrected p-value** |
| --- | --- | --- | --- | --- | --- | --- |
| **Shannon** | Control | D0 | D28 | 8.0 | 0.688 | 0.931 |
|  |  | D28 | D56 | 9.0 | 0.844 | 0.931 |
|  |  | D56 | D84 | 3.0 | 0.156 | 0.455 |
|  |  | D84 | DX | 0.0 | 0.031 | 0.333 |
| **Observed features** |  | D0 | D28 | 0.0 | 0.031 | 0.333 |
|  |  | D28 | D56 | 7.0 | 0.563 | 0.931 |
|  |  | D56 | D84 | 10.0 | 1.000 | 1.000 |
|  |  | D84 | DX | 1.0 | 0.063 | 0.333 |
| **Pielou’s evenness** |  | D0 | D28 | 4.0 | 0.219 | 0.538 |
|  |  | D28 | D56 | 9.0 | 0.844 | 0.931 |
|  |  | D56 | D84 | 1.0 | 0.063 | 0.333 |
|  |  | D84 | DX | 1.0 | 0.063 | 0.333 |
| **Faith’s PD** |  | D0 | D28 | 2.0 | 0.094 | 0.333 |
|  |  | D28 | D56 | 4.0 | 0.219 | 0.538 |
|  |  | D56 | D84 | 5.0 | 0.313 | 0.714 |
|  |  | D84 | DX | 2.0 | 0.094 | 0.333 |
| **Shannon** | Probiotic | D0 | D28 | 2.0 | 0.094 | 0.333 |
|  |  | D28 | D56 | 8.0 | 0.688 | 0.931 |
|  |  | D56 | D84 | 8.0 | 0.688 | 0.931 |
|  |  | D84 | DX | 7.0 | 0.563 | 0.931 |
| **Observed features** |  | D0 | D28 | 0.0 | 0.031 | 0.333 |
|  |  | D28 | D56 | 7.0 | 0.893 | 0.952 |
|  |  | D56 | D84 | 9.0 | 0.844 | 0.931 |
|  |  | D84 | DX | 9.0 | 0.844 | 0.931 |
| **Pielou’s evenness** |  | D0 | D28 | 7.0 | 0.563 | 0.931 |
|  |  | D28 | D56 | 9.0 | 0.844 | 0.931 |
|  |  | D56 | D84 | 9.0 | 0.844 | 0.931 |
|  |  | D84 | DX | 7.0 | 0.563 | 0.931 |
| **Faith’s PD** |  | D0 | D28 | 3.0 | 0.156 | 0.455 |
|  |  | D28 | D56 | 10.0 | 1.000 | 1.000 |
|  |  | D56 | D84 | 9.0 | 0.844 | 0.931 |
|  |  | D84 | DX | 9.0 | 0.844 | 0.931 |

**Table 3:** Mixed Linear Model Regression Results for Alpha Diversity metric Shannon

| **Mixed Linear Model Regression Results** | | | | | | |
| --- | --- | --- | --- | --- | --- | --- |
| Model: | MixedLM | Dependent Variable: | | shannon_entropy | | |
| No. Observations: | 60 | Method: | | REML | | |
| No. Groups: | 12 | Scale: | | 0.0424 | | |
| Min. group size: | 5 | Log-Likelihood: | | -0.9044 | | |
| Max. group size: | 5 | Converged: | | Yes | | |
| Mean group size: | 5.0 |  |  |  |  |  |
|  | **Coef.** | **Std.Err.** | **z** | **P>\|z\|** | **[0.025** | **0.975]** |
| Intercept | 7.435 | 0.093 | 79.852 | 0.000 | 7.252 | 7.617 |
| group[T.probiotic] | -0.114 | 0.132 | -0.865 | 0.387 | -0.372 | 0.144 |
| time | 0.037 | 0.027 | 1.381 | 0.167 | -0.015 | 0.089 |
| time:group[T.probiotic] | 0.045 | 0.038 | 1.210 | 0.226 | -0.028 | 0.119 |
| Group Var | 0.005 | 0.033 |  | | | |

**Table 4:** Mixed Linear Model Regression Results for Alpha Diversity metric Observed Features

| **Mixed Linear Model Regression Results** | | | | | | |
| --- | --- | --- | --- | --- | --- | --- |
| Model: | MixedLM | Dependent Variable: | | observed_features | | |
| No. Observations: | 60 | Method: | | REML | | |
| No. Groups: | 12 | Scale: | | 1063.5426 | | |
| Min. group size: | 5 | Log-Likelihood: | | -282.1038 | | |
| Max. group size: | 5 | Converged: | | Yes | | |
| Mean group size: | 5.0 |  |  |  |  |  |
|  | **Coef.** | **Std.Err.** | **z** | **P>\|z\|** | **[0.025** | **0.975]** |
| Intercept | 363.150 | 13.965 | 26.004 | 0.000 | 335.779 | 390.521 |
| group[T.probiotic] | -9.133 | 19.750 | -0.462 | 0.644 | -47.842 | 29.575 |
| time | 11.650 | 4.210 | 2.767 | 0.006 | 3.398 | 19.902 |
| time:group[T.probiotic] | 4.167 | 5.954 | 0.700 | 0.484 | -7.503 | 15.837 |
| Group Var | 0.236 | 5.061 |  | | | |

**Table 5:** Mixed Linear Model Regression Results for Alpha Diversity metric Pielou’s Evenness

| **Mixed Linear Model Regression Results** | | | | | | |
| --- | --- | --- | --- | --- | --- | --- |
| Model: | MixedLM | Dependent Variable: | | pielou_evenness | | |
| No. Observations: | 60 | Method: | | REML | | |
| No. Groups: | 12 | Scale: | | 0.0003 | | |
| Min. group size: | 5 | Log-Likelihood: | | 137.2323 | | |
| Max. group size: | 5 | Converged: | | Yes | | |
| Mean group size: | 5.0 |  |  |  |  |  |
|  | **Coef.** | **Std.Err.** | **z** | **P>\|z\|** | **[0.025** | **0.975]** |
| Intercept | 0.875 | 0.008 | 110.038 | 0.000 | 0.860 | 0.891 |
| group[T.probiotic] | -0.010 | 0.011 | -0.910 | 0.363 | -0.032 | 0.012 |
| time | -0.000 | 0.002 | -0.167 | 0.868 | -0.005 | 0.004 |
| time:group[T.probiotic] | 0.004 | 0.003 | 1.222 | 0.222 | -0.002 | 0.010 |
| Group Var | 0.000 | 0.003 |  | | | |

**Table 6**: Differences between groups for Beta Diversity, Weighted UniFrac Metric in each timepoint - PERMANOVA (9999 permutations) results with Benjamini-Hochberg correction

| Time Point | p-value | corrected p-value |
| --- | --- | --- |
| D0 | 0.0124 | 0.062 |
| D28 | 0.4132 | 0.9909 |
| D56 | 0.7927 | 0.9909 |
| D84 | 0.9955 | 0.9955 |
| DX | 0.6611 | 0.9909 |

**Table 7**: Mixed Linear Model Regression Results for Acetic Acid

| Mixed Linear Model Regression Results | | | | | | |
| --- | --- | --- | --- | --- | --- | --- |
| Model: | MixedLM | Dependent Variable: | | Acetic Acid | | |
| No. Observations: | 60 | Method: | | REML | | |
| No. Groups: | 12 | Scale: | | 2.0756 | | |
| Min. group size: | 5 | Log-Likelihood: | | -108.1727 | | |
| Max. group size: | 5 | Converged: | | Yes | | |
| Mean group size: | 5.0 |  |  |  |  |  |
|  | Coef. | Std.Err. | z | P>\|z\| | [0.025 | 0.975] |
| Intercept | 0.052 | 0.626 | 0.083 | 0.934 | -1.175 | 1.279 |
| group[T.probiotic] | 0.572 | 0.885 | 0.646 | 0.518 | -1.164 | 2.307 |
| time | 1.106 | 0.186 | 5.946 | 0.000 | 0.741 | 1.471 |
| time:group[T.probiotic] | -0.414 | 0.263 | -1.573 | 0.116 | -0.929 | 0.102 |
| Group Var | 0.069 | 0.166 |  | | | |

**Table 8:** Mixed Linear Model Regression Results for Propionic Acid

| Mixed Linear Model Regression Results | | | | | | |
| --- | --- | --- | --- | --- | --- | --- |
| Model: | MixedLM | Dependent Variable: | | Propionic Acid | | |
| No. Observations: | 60 | Method: | | REML | | |
| No. Groups: | 12 | Scale: | | 0.3778 | | |
| Min. group size: | 5 | Log-Likelihood: | | -59.7010 | | |
| Max. group size: | 5 | Converged: | | Yes | | |
| Mean group size: | 5.0 |  |  |  |  |  |
|  | Coef. | Std.Err. | z | P>\|z\| | [0.025 | 0.975] |
| Intercept | -0.089 | 0.263 | -0.338 | 0.736 | -0.605 | 0.427 |
| group[T.probiotic] | 0.303 | 0.372 | 0.814 | 0.415 | -0.426 | 1.033 |
| time | 0.400 | 0.079 | 5.041 | 0.000 | 0.244 | 0.555 |
| time:group[T.probiotic] | -0.178 | 0.112 | -1.590 | 0.112 | -0.398 | 0.041 |
| Group Var | 0.000 | 0.061 |  | | | |

**Table 9:** Mann-Whitney test results for probiotic and control groups for IgA at each time point with Benjamini-Hochberg correction

| Timepoint | p-value | corrected p-value |
| --- | --- | --- |
| D0 | 0.310 | 0.818 |
| D28 | 0.818 | 0.818 |
| D56 | 0.818 | 0.818 |
| D84 | 0.699 | 0.818 |
| DX | 0.394 | 0.818 |

**Table 10:** Wilcoxon test results for control and probiotic groups for IgA at each time point with Benjamini-Hochberg correction

| Probiotic Group | | | Control Group | | |
| --- | --- | --- | --- | --- | --- |
| Comparison | p-value | corrected  p-value | Comparison | p-value | corrected  p-value |
| D0 vs. D28 | 0.688 | 0.764 | D0 vs. D28 | 0.313 | 0.729 |
| D0 vs. D56 | 0.438 | 0.729 | D0 vs. D56 | 0.438 | 0.729 |
| D0 vs. D84 | 0.688 | 0.764 | D0 vs. D84 | 0.063 | 0.625 |
| D0 vs. DX | 0.219 | 0.547 | D0 vs. DX | 0.313 | 0.729 |
| D28 vs. D56 | 0.219 | 0.547 | D28 vs. D56 | 0.844 | 0.844 |
| D28 vs. D84 | 0.313 | 0.625 | D28 vs. D84 | 0.156 | 0.729 |
| D28 vs. DX | 0.563 | 0.764 | D28 vs. DX | 0.438 | 0.729 |
| D56 vs. D84 | 0.844 | 0.844 | D56 vs. D84 | 0.844 | 0.844 |
| D56 vs. DX | 0.063 | 0.547 | D56 vs. DX | 0.844 | 0.844 |
| D84 vs. DX | 0.156 | 0.547 | D84 vs. DX | 0.844 | 0.844 |

**Table 11**: Mann-Whitney test results for probiotic and control groups for EPG at each time point with Benjamini-Hochberg correction

| Timepoint | p-value | corrected p-value |
| --- | --- | --- |
| D0 | 0.676 | 0.739 |
| D28 | 0.568 | 0.739 |
| D56 | 0.739 | 0.739 |
| D84 | 0.625 | 0.739 |
| DX | 0.446 | 0.739 |

**Table 12:** Wilcoxon test results for control and probiotic groups for EPG at each time point with Benjamini-Hochberg correction

| Probiotic Group | | | Control Group | | |
| --- | --- | --- | --- | --- | --- |
| Comparison | p-value | corrected  p-value | Comparison | p-value | corrected  p-value |
| D0 vs. D28 | 0.068 | 0.136 | D0 vs. D28 | 0.068 | 0.544 |
| D0 vs. D56 | 0.102 | 0.171 | D0 vs. D56 | 0.180 | 0.599 |
| D0 vs. D84 | 0.043 | 0.108 | D0 vs. D84 | 0.109 | 0.544 |
| D0 vs. DX | 0.144 | 0.206 | D0 vs. DX | 1.000 | 1.000 |
| D28 vs. D56 | 0.581 | 0.581 | D28 vs. D56 | 0.715 | 0.794 |
| D28 vs. D84 | 0.042 | 0.108 | D28 vs. D84 | 0.465 | 0.741 |
| D28 vs. DX | 0.581 | 0.581 | D28 vs. DX | 0.273 | 0.683 |
| D56 vs. D84 | 0.043 | 0.108 | D56 vs. D84 | 0.593 | 0.741 |
| D56 vs. DX | 0.465 | 0.581 | D56 vs. DX | 0.593 | 0.741 |
| D84 vs. DX | 0.043 | 0.108 | D84 vs. DX | 0.414 | 0.741 |
